# The Effective Role of Adding Letrozole to Methotrexate in the Management of Tubal Ectopic Pregnancies, a Randomized Clinical Trial

**DOI:** 10.22037/ijpr.2021.115659.15461

**Published:** 2021

**Authors:** Zahra Rezaei, Marjan Ghaemi, Elham Feizabad, Behnaz Ghavami, Firoozeh Akbari Asbagh, Fatemeh Davari Tanha, Mahbod Ebrahimi, Zahra Khalaj Sereshki

**Affiliations:** a *Yas Hospital, Tehran University of Medical Sciences, Tehran, Iran.*; b *Vali-e-Asr Reproductive Health Research Center, Tehran University of Medical Sciences, Tehran, Iran. *; c *Arash Women Hospital, Tehran University of Medical Sciences, Tehran, Iran.*

**Keywords:** Letrozole, Ectopic Pregnancy, β-hCG, Methotrexate, Efficacy, Safety.

## Abstract

Ectopic pregnancy (EP) is considered a main reproductive health challenge. According to the side effects of using methotrexate (MTX), it is rational to find safer drugs in the management of EP. This randomized controlled trial aimed to evaluate the efficacy and safety of adding letrozole to the single-dose MTX in the management of EPs. This study was conducted in an academic hospital affiliated to Tehran University of Medical Sciences. Women with EP and stable vital signs with β-hCG levels ≤3500 were assigned randomly to receive MTX + placebo or MTX + letrozole. The regression pattern of β-hCG, need for further surgery, and potential side effects were compared between groups. A total of 90 women were assigned equally to the study groups and were matched in age, body mass index (BMI), serum biochemistry, and primary levels of β-hCG. No drug-related side effects were observed in groups. The rates of further surgery (p = 0.614) and second dose of MTX (p = 0.809) were not significant between groups. In the MTX + placebo group, we observed a minor increase in β-hCG levels on day 4 followed by a decreasing pattern on days 7 and 14. But, in MTX + letrozole group, a decreasing pattern in β-hCG levels from day 1 through day 14 was perceived. The results support using MTX + letrozole to treat stable women diagnosed with tubal EP as a safe and efficient method. Further studies are required to evaluate letrozole alone as an alternative therapy in EPs.

## Introduction

Ectopic pregnancies (EPs) are the potential life threatening conditions that occur in 1-2% of all pregnancies (1), and 98% of them take place in the fallopian tubes (2). This condition can be managed surgically (salpingectomy or salpingostomy), medically with methotrexate (MTX), or expectantly (3). MTX, a dihydrofolate reductase inhibitor and folic acid antagonist, has been widely used as the first-line medical treatment for early unruptured EPs as single or multiple-dose regimens, with success rates up to 90% (4). 

However, there are several problems with using this drug, some of which include serious side effects and specific contraindications. Indeed, long treatment periods (in most cases), long-term hospitalization, and the need for frequent doses of this drug affect the patients’ quality of life. Also, the need for emergency or non-emergency surgical treatment is another concern in the medical or expectant management of EP (3). 

Letrozole is a reversible third-generation aromatase inhibitor administered in medical abortion lonely or in combination with other drugs such as misoprostol or MTX (5). This agent can reduce estradiol levels in early pregnancy up to 95-99% (6), and also it may disrupt the physiological functions of progesterone needed to maintain pregnancy (7). 

The use of letrozole in the treatment of EP has not been widely studied (7), and it is hypothesized that the addition of letrozole may increase the success rate of medical treatment of EP. The purpose of this study was to verify letrozole + MTX as a treatment option among patients with tubal EP. Furthermore, this trial aimed to investigate the short-term efficacy and safety of this combination therapy and compare it with MTX administration alone. 

## Experimental

*Study setting*

This prospective and blinded randomized clinical trial was conducted in an academic referral hospital affiliated to Tehran University of Medical Sciences from August 2019 to October 2020. 

*Ethical considerations*

This study was approved by review boards of Tehran University of Medical Sciences by reference number: IR.TUMS.MEDICINE.REC.1399.070 and registered as a clinical trial by reference number IRCT20130808014301N2. The protocol of the study was designed according to the ethical principles of the Declaration of Helsinki. All participants agreed to participate in the study, and written informed consent was obtained from all participants. 

*Eligibility criteria*

Inclusion criteria were:

Tubular EP as an inappropriate increase in β-hCG levels less than or equal to 63% increase within 48 h

Age between 18 and 45 years

A β-hCG levels ≤3500 

Absence of fetal heart rate

The average diameter of the adnexal mass ≤3.5 cm

Stable hemodynamic condition

Absence of significant abdominal pain

No history of allergic reactions to MTX

No history of allergic reactions to letrozole and other aromatase inhibitors

Exclusion criteria were:

Dissatisfaction with MTX or letrozole administration

Methadone use

Significant free fluid in the pelvis

Any known liver disorder or abnormal liver enzyme levels (AST or ALT) 

Any known renal disorder or impaired renal function tests (abnormal creatinine)

Heterotopic pregnancy

*Randomization*

After confirming the eligibility criteria, participants were randomly assigned to MTX + placebo and MTX + letrozole groups in a 1:1 allocation by a computer‐generated simple randomization list. The allocation sequence was kept blinded to the recruiters and local staff. The stratification factors to be balanced across treatment groups were age and BMI. The patient, the prescribing nurse, the data collector, and the statistical analyst were unaware of the intervention. 

*Patient setting*

At the beginning of the study, preliminary serum biochemistry markers such as hematological, hepatic, and creatinine were obtained and repeated at the end of the treatment period. The serum β-hCG titration was checked with the same laboratory kit on days 1, 4, 7, and 14 of treatment.

After confirming tubal EP diagnosis. On the same day, a single dose of intramuscular MTX (50 mg/m^2^) by Ebewe Pharma, Bulgaria was ordered in both groups. The MTX + placebo group took two placebo tablets every 12 hours for seven days from the day of MTX administration; while the MTX + letrozole group received two letrozole tablets (Abureyhan Co., Iran, 2.5 milligram letrozole tablets) every 12 hours for seven days from the day of MTX administration. 

If the decrease in β-hCG level between days 4 and 7 was less than 15%, the second dose of intramuscular MTX (50 mg/m^2^) was administered. After day 7, the β-hCG level was checked weekly. All patients were hospitalized at least in the first week of intervention. They were also advised to increase their fluid intake and avoid exposure to sunlight. They were informed of the common side effects of MTX and warned to avoid alcohol, non‐steroidal anti‐inflammatory drugs and aspirin. 

Patients with severe pain were further evaluated with transvaginal ultrasonography. Patients with findings of hemoperitoneum by ultrasound or any hemodynamic changes which may accompany a tubal rupture underwent emergency surgery. 

*Criteria for discontinuation of trial treatment*

Severe adverse effects which may be related to the study medication, severe pain with findings of hemoperitoneum by transvaginal ultrasonography, hemodynamic changes, or unstable general condition that led to emergency management were the criteria for discontinuation of the trial.

*Outcome measures*

During and at the end of the study, the need for urgent surgical intervention and instability of patients’ hemodynamic as well as safety and efficacy of two regimens and their effect on the course of serum β-hCG titration were evaluated. 

*Statistical analysis*

The statistical analyses were done using Statistical Package for the Social Sciences (SPSS) version 24.0 (IBM, New York, USA). A *P*-value of less than 0.05 was determined as the level of statistical significance. We used Independent *T*-test and repeated measures to assess differences in means. A Chi-square and Fisher’s Exact test were applied to evaluate differences in proportions.

## Results

**Figure 1:**
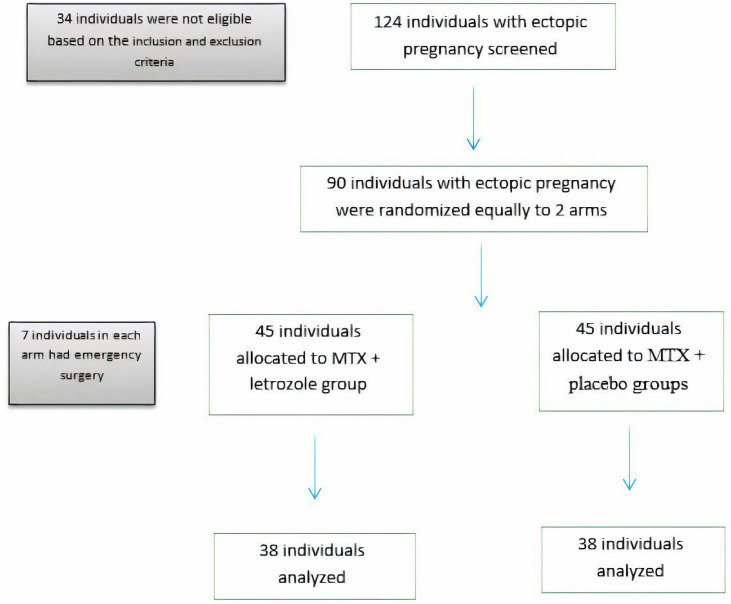
The CONSORT flowchart of the participant's recruitment

A total of 124 individuals were screened, 90 of whom met the eligibility criteria and were randomized in two equal groups (45 cases in each arm). None of the women declined the trial intervention after randomization (Figure 1).

The mean age of women in the MTX + letrozole and MTX + placebo groups were 32.13 ± 4.45 and 31.97 ± 4.58 years old, respectively, with no significant difference (*p* = 0.871). Furthermore, the two groups were similar in age and baseline complete blood count (CBC), serum biochemistry, and β-hCG level (Table 1).

The CBC and serum biochemistry characteristics of patients on the seventh day of the study were not significantly different among groups, and the details are shown in Table 2. Twelve cases in MTX + letrozole group and 11 cases in MTX + placebo group needed administration of the second dose of MTX with no difference (*p* = 0.809) between groups. Also, the surgery rate (*p* = 0.614) was not significantly different among groups (7 cases in each group).

As shown in Table 3, the β-hCG levels declined significantly (*p* < 0.001, repeated measures test) in all participants from day one to 14th day of study. Furthermore, β-hCG levels between the two groups did not have any significant difference (*p* = 0.270). 

**Figure 2:**
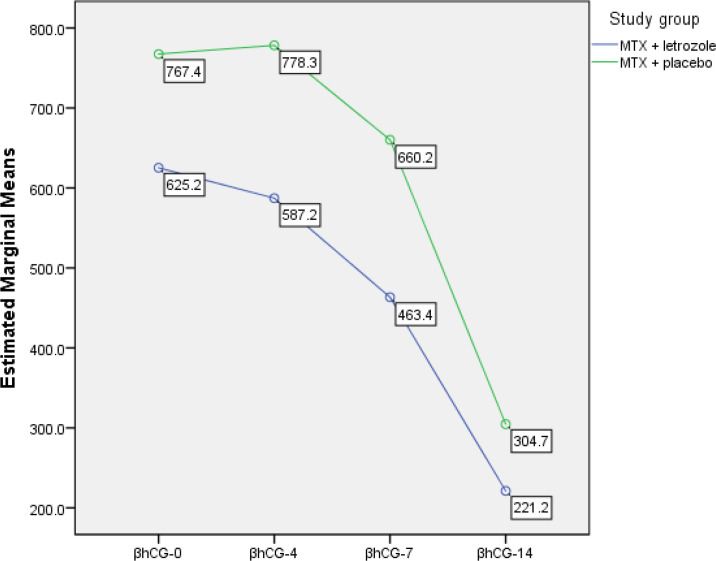
The pattern of β–hCG level changes from day one to the 14th day of study (38 participants in each group)

In the MTX + placebo group, we observed a minor increase in β-hCG levels from day 1 through day 4 followed by a decreasing pattern at days 7 and 14. But, in MTX + letrozole group, a decreasing pattern in β-hCG levels from day 1 through day 14 was observed (Figure 2).

**Table 1:** Baseline CBC and serum biochemistry characteristics in both groups

**Variables**	**Mean ± SD**		***p***
	**MTX + letrozole**	**MTX + placebo**	
Hemoglobin (g/dL)	12.43 ± 1.11	12.26 ± 1.2	0.480
WBC (WBCs/mL)	7771.77 ± 2020.59	7390.66 ± 1743.51	0.341
Platelets (platelets/mL)	242.44 ± 53.51	257.08 ± 49.11	0.180
AST (IU/L)	20.35 ± 4.4	23.08 ± 8.58	0.062
ALT (IU/L)	15.77 ± 6.82	18.64 ± 9.78	0.111
Creatinine (mg/dL)	1.19 ± 2.56	0.82 ± 0.1	0.338

MTX: methotrexate; WBC: white blood cells; AST: Aspartate aminotransferase; ALT: alanine aminotransferase;

β–hCG: beta human chorionic gonadotropin; SD: standard deviation.

**Table 2:** CBC and serum biochemistry characteristics on seventh day of study in both groups

**Variables**	**Mean ± SD**		***p***
	**MTX + letrozole**	**MTX + placebo**	
Hemoglobin (g/dL)	12.41 ± 1.09	12.20 ± 1.12	0.385
WBC (WBCs/mL)	7662.88 ± 2075.18	7252.66 ± 1699.37	0.308
Platelets (platelets/mL)	243.93 ± 52.12	260.11 ± 49.51	0.135
AST (IU/L)	18.11 ± 4.91	20.8 ± 8.03	0.059
ALT (IU/L)	17.35 ± 5.87	17.75 ± 9.44	0.810
Creatinine (mg/dL)	0.84 ± 0.11	0.82 ± 0.08	0.270

MTX: methotrexate; WBC: white blood cells; AST: Aspartate aminotransferase; ALT: alanine aminotransferase; SD: standard deviation.

**Table 3:** The serum β–hCG level changes overall and between two groups

**Variables**		**Mean ± SD**	**95% Confidence Interval**		***p***
			**Lower Bound**	**Upper Bound**	
Overall	β –hCG 1	696.31 ± 73.29	550.29	842.34	<0.001
	β –hCG 4	682.75 ± 84.30	515.32	850.19	
	β –hCG 7	561.81 ± 81.78	398.86	724.78	
	β –hCG 14	262.97 ± 50.23	162.89	363.07	
MTX + letrozole	β –hCG 1	625.21 ± 103.64	418.70	831.73	0.270
	β –hCG 4	587.23 ± 118.83	350.45	824.02	
	β –hCG 7	463.42 ± 115.66	232.97	693.88	
	β –hCG 14	221.21 ± 71.04	79.66	362.76	
MTX + placebo	β –hCG 1	767.41 ± 103.64	560.90	973.93	
	β –hCG 4	778.27 ± 118.83	541.49	1015.06	
	β –hCG 7	660.21 ± 115.66	429.75	890.67	
	β –hCG 14	304.74 ± 71.04	163.19	446.30	

SD: standard deviation.

## Discussion

EP is considered a main reproductive health challenge that may serve as a life-threatening emergency in obstetrics and remains a significant cause of maternal morbidity and mortality, especially in less developed countries (11). MTX is used as a routine for the medical treatment of EPs. According to the known side effects of using this drug which is a chemotherapy agent, management of this condition with minimal complications and high safety seems reasonable. This study showed the success of MTX + letrozole in the treatment of EP with no significant side effects. In the MTX + placebo group, there was a minor increase in β-hCG levels on day 4. This may be due to continued β-hCG production by syncytiotrophoblasts despite cessation of production by cytotrophoblasts that is a common phenomenon (12). This rise did not occur in MTX + letrozole group that might be due to the letrozole effect. 

The usefulness of letrozole for the termination of pregnancy has been demonstrated in several animal and human studies (16). Letrozole as an aromatase inhibitor binds to P450 cytochrome reversibly and prevents estrogen production by aromatase (13). P450 cytochrome converts androgens to estrogen, which is necessary for the continuation of pregnancy (14). Tracy *et al.* demonstrated the beneficial effects of taking letrozole for three days before misoprostol administration and reported a complete abortion rate of 95% (15). Also, a systematic review of seven studies using letrozole in abortion showed that adding letrozole to routine abortion treatments was associated with a high success rate (16). 

The studies on the effect of letrozole in the management of EP are scarce (7). Currently there is one trial that is registered in clinical trial.gov that is not recruited yet. Mitwally et al. published their study at the American Society for Reproductive Medicine (ASRM) Congress in 2019 and compared letrozole and MTX in treating EPs and showed equal efficacy in both groups. MTX treatment arm was associated with significantly higher levels of liver enzymes and lower platelet counts (7).

In the mentioned study, β-hCG levels decreased more rapidly in the letrozole group than in the MTX group and were lower in the letrozole group three months after treatment; however, this difference was not statistically significant (7). This is in line with our study after 2 weeks follow up that the β-hCG levels decrease much better in MTX + letrozole group in comparison with MTX + placebo group. The differences of this study with our work were the larger sample size in our study. Also, we administered MTX in both groups and added letrozole to one study arm. In Mitwally study, some patients underwent emergency surgery due to their unstable vital signs and emergency status, but in ours, none of the patients needed emergency surgery. This difference can be explained by the fact that we did not exclude any patients from routine management (MTX), and perhaps that is why the patients needed no emergency actions.

Letrozole is a safe drug and is preferred over the MTX, which is used as a chemotherapeutic agent. It could be a reasonable option for the medical treatment of EP. The strength of our study was using a randomization method to decrease confounding factors and acceptable sample size. The limitation of our study was a short follow-up duration. Further studies with longer follow-up periods and larger sample sizes are required to evaluate letrozole alone as an alternative therapy in EP.

## Conclusion

 The results of our study support using MTX + letrozole for the treatment of clinically stable women diagnosed with tubal EP as a safe and more efficient alternative than MTX + placebo.

## Author contributions

 Z.R.: Design of the work 

M.G: Drafting the manuscript

E.F.: Data analysis

F.A.S: Manuscript editing, Interpretation of data

F.D.T: Manuscript editing, Interpretation of data

M.E: Interpretation of data 

Z.K.S**: **Data gathering, Design of the work 
